# Evaluation of by-products from agricultural, livestock and fishing industries as nutrient source for the production of proteolytic enzymes

**DOI:** 10.1016/j.heliyon.2023.e20735

**Published:** 2023-10-10

**Authors:** Alisson Sisa, Cristina Sotomayor, Lucía Buitrón, Joaquín Gómez-Estaca, Oscar Martínez-Alvarez, Mauricio Mosquera

**Affiliations:** a Department of Food Science and Biotechnology, Escuela Politécnica Nacional, Quito, P.O. Box 17-01-2759, Ecuador; b Institute of Food Science, Technology and Nutrition (ICTAN-CSIC), 6 José Antonio Novais St., 28040, Madrid, Spain

**Keywords:** Proteolytic enzymes, Agro-industrial byproducts, *Bacillus subtilis*, Enzymatic activity, Culture media, Purification, Sustainable production

## Abstract

This study presents an approach that utilizes low-value agro-industrial by-products as culture media for producing high-value proteolytic enzymes. The objective was to assess the impact of six agro-industrial by-products as culture media on the production of proteolytic enzymes. Bacillus subtilis strains, confirmed through comprehensive biochemical, morphological, and molecular analyses, were isolated and identified. Enzymatic activity was evaluated using azocasein and casein substrates, and the molecular sizes of the purified extract components were determined. The results demonstrated that the isolated bacteria exhibited higher metabolic and enzymatic activity when cultured in media containing 1 % soybean oil cake or feather meal. Furthermore, higher concentrations of the culture media were found to hinder the production of protease. Optimal protease synthesis on soybean oil cake and feather meal media was achieved after 4 days, using both the azocasein and casein methods. Semi-purification of the enzymatic extract obtained from Bacillus subtilis in feather meal and soybean oil cake resulted in a significant increase in azocaseinolytic and caseinolytic activities. Gel electrophoresis analysis revealed multiple bands in the fractions with the highest enzymatic activity in soybean oil cake, indicating the presence of various enzymes with varying molecular sizes. These findings highlight the potential of utilizing low-value agro-industrial by-products as efficient culture media for the sustainable and economically viable production of proteolytic enzymes with promising applications in various industries.

## Highlights

• Protease-producing bacteria can be effectively isolated from the environment.
• Agro-industrial byproducts can serve as suitable growth media for microbial cultures.
• Bacillus subtilis demonstrates a high potential for protease production.
• Feather meal and palm soybean oil-cake are effective media for bacterial growth.

## 1 Introduction

Enzymes have attracted the attention of researchers due to their role in analytical, physiological, and industrial applications. They also enable more efficient use of raw materials, saving water and energy, and their demand is therefore constantly increasing, driven by the growing need for sustainable solutions [1]. Proteolytic enzymes also known as proteases or peptidases, break down the peptide bonds of proteins to produce smaller chains. Proteases represent around 60 % of total enzyme sales and are one of the most important groups of industrial enzymes with wide distribution in nature [2].

Microorganisms have emerged as the primary source of proteases, surpassing plant and animal sources, owing to their superior production capabilities. They offer distinct advantages, including ease of cultivation and higher production yields, making them more efficient for protease synthesis. Additionally, the utilization of economically available culture media from agro-food industry waste significantly reduces production costs. This strategic utilization of microorganisms and waste-derived culture media represents a sustainable and economically viable approach to protease production in various industries [3]. In recent years, there has been an increasing in the use of solid waste for protease production, including the utilization of dairy waste as a by-product of the dairy industry. These wastes are rich in nutrients, including proteins and amino acids, making them an interesting option for protease production due to their favorable nutritional properties that promote both microbial growth and enzyme synthesis [4]. Food processing generates numerous by-products, including dairy waste from the processing of milk into dairy products such as cheese, yogurt, and butter [5], and various food waste matrices such as wheat bran [6], potato peels [7], amaranth flour [8], soybean meal [9], feather meal [10], shrimp shells [11] and cow dung [12]. These waste materials have garnered significant attention in the search for sustainable solutions, as their utilization holds great promise for addressing both waste management challenges and the production of valuable proteases.

The most industrially exploited bacterial proteases are from the genus *Bacillus* [13]. These microorganisms grow rapidly in short fermentation times and produce high levels of enzymes with significant proteolytic activity [14]. Several protease-producing *Bacillus* species have been reported, such as *B. alveayuensis*, isolated from shrimp and crab shell dust [15], *B. cereus* isolated from buffalo hide [16], *B. mojavensis*, isolated from soil samples [17], and *B. subtilis* isolated from the soil of a milk processing plant [18]. It is worth noting that *Bacillus subtilis* is a prominent bacterium used extensively for the industrial production of proteolytic enzymes. This microorganism is of great due to its exceptional tolerance and adaptability to adverse environmental conditions [13].

The aim of this work was to evaluate the effect of culture media, specifically four different agricultural, livestock and fishery by-products, on the production of a proteolytic extract from wild bacteria in the laboratory environment.

## 2 Materials and methods

### 2.1 Materials

The bovine gelatin utilized in this study was sourced from a local chemical reagent store (La Casa del Químico, Quito - Ecuador). Nutrient media of both animal and vegetable origin, including feather meal, fish meal, palm kernel oil-cake, soybean oil-cake, and rice polish, were procured in bulk from the province of Santo Domingo (Ecuador). Additionally, blood meal was donated by the metropolitan slaughterhouse of Quito.

Analytical grade reagents obtained for the experiment comprised Natamycin (Mayasan AS), BHI-Agar (Remel), BHI (Becton, Dickinson and Company), potassium phosphate monobasic and dibasic (Fisher Scientific), sodium chloride (Novachem), dextrose, ammonium sulfate (CIA Chemist House), Sephadex G-100 resin (Sigma Aldrich), trichloroacetic acid (Analar), casein (Merck), Tris-HCl (Invitrogen), sodium hydroxide (Merck KGaA), mercaptoethanol, SDS, azocasein (Sigma Aldrich), glycerol (Macron), bromophenol brilliant blue, Coomassie brilliant blue, acrylamide, and bisacrylamide (Bio-Rad).

### 2.2 Isolation and identification of the grown bacteria from the laboratory environment

#### 2.2.1 Isolation of the bacteria

Bovine gelatin was utilized as the culture medium for cultivating the wild microorganism from the laboratory environment. Initially, 100 mL of distilled water was heated to boiling with continuous stirring. Once the water reached a boiling point, 3 g of gelatin was introduced. Subsequently, to prevent the growth of fungi and yeasts, 0.5 g of natamycin was added after the complete dissolution of the gelatin [19]. The mixture underwent sterilization in an autoclave and was then cooled to 37 °C. The solution was subsequently left uncovered in an oven at 37 °C for one week to facilitate incubation. After this incubation period, samples of the colonies that had developed in the medium were inoculated onto a plate [20]. The plate was then incubated at 37 °C for 48 h, following which it was transferred to fresh plates until a pure colony of microorganisms was successfully isolated.

#### 2.2.2 Biochemical and morphological identification of the isolated bacteria

Tests to determine the biochemical and morphological characteristics of the isolated strain were carried out according to Bergey's Manual of Systematic Bacteriology [21] and included: Gram staining, urea agar base (based on urease activity), Simons citrate agar (based on citrate metabolism), motility (presence of flagellum), indole (based on tryptophan hydrolysis) and H_2_S production.

#### 2.2.3 Molecular identification of isolated bacteria

For the molecular identification of the microorganism, DNA extractions were performed, ensuring the retrieval of high-quality genetic material. The 16S region, a highly conserved region of the bacterial genome, was then amplified using the polymerase chain reaction (PCR) technique. This targeted amplification of the 16S region facilitated the acquisition of specific genetic markers crucial for accurate identification and classification of the microorganism. Finally, gene sequencing was employed as the concluding step in the process, enabling the determination of the precise DNA sequence and providing valuable insights into the microorganism's taxonomic position. This comprehensive molecular approach yielded reliable and detailed information for the identification and classification of the microorganism.

The method for identifying the isolated bacterium is detailed as follows:

##### 2.2.3.1 DNA extraction

Extraction of genetic material was performed by adapting the techniques described by Raeder & Broda (1985) with certain modifications [22]. Samples were centrifuged at 5000×*g* for 10 min, and to the pellet, 1 mL of TES buffer (Sucrose, EDTA, Tris-HCl; pH 8) was added before centrifuging again under the same conditions. The pellet was resuspended in 180 μL of Genomic Digestion buffer, followed by the addition of 25 μL of proteinase K, and incubated at 55 °C for 1 h. After incubation, the samples were centrifuged at 5000×*g* for 10 min, and 200 μL of Genomic Lysis buffer (lysozyme, mutanolysin, and TET buffer) along with 200 μL of 100 % ethanol were added to the supernatant. The mixture was then centrifuged, and the supernatant was collected. Subsequently, 500 μL of Wash buffer was added and centrifuged at 10,000×*g* for 1 min. The supernatant was treated with 500 μL of Wash buffer 2 and centrifuged again for 3 min. After the allotted time, 100 μL of water was added and incubated at room temperature for 2 min, followed by centrifugation (10,000×*g* for 1.5 min). Lastly, 1.5 μL of RNase was added and incubated at 37 °C for 10 min. Finally, 8 μL of the sample was taken for DNA concentration measurement and stored at −20 °C. The presence or absence of DNA was determined using 1 % agarose gel electrophoresis.

##### 2.2.3.2 Amplification of the 16S region

The amplification of the 16S region was carried out using the polymerase chain reaction technique (PCR). For this, 1 μL of extracted DNA sample, 12.5 μL of Taq polymerase (from [enzyme source]), 0.5 μL each of forward primer 27F (5′AGAGTTTGATCCTGGCTCA 3′) and reverse primer 1492R (5′GGTTACCTTGTTACGACTT 3′), and 10 μL of nuclease-free water were combined, resulting in a final reaction volume of 25 μL. The PCR reaction cycles were performed as follows: initial denaturation at 94 °C for 5 min, followed by 30 cycles of denaturation at 94 °C for 1 min, annealing at 53 °C for 1 min, and extension at 72 °C for 1 min. A final extension step was carried out at 72 °C for 10 min, followed by maintenance at 4 °C [23]. To confirm the success of the amplification, a 1 % agarose gel electrophoresis was performed. The amplified PCR products were forwarded to Macrogen Inc., a renowned biotechnology company based in South Korea, for precise identification. Macrogen Inc. is distinguished for its exceptional expertise and unwavering commitment to providing highly accurate and reliable results.

### 2.3 Identification of the optimal culture medium

To determine the most suitable culture medium for the isolated bacteria, six protein sources were investigated at various concentrations. The study focused on both plant-derived sources, including palm kernel oil cake, soy oil cake, and rice polish, and animal-derived sources, such as feather meal, fish meal, and bovine blood meal. Each protein source was evaluated individually to assess its impact on the growth and enzymatic activity of the bacteria. Different concentrations of the culture medium, ranging from 1 % to 4 % w/v, were tested to understand the influence of varying nutrient levels on enzymatic activity. The enzymatic activity was quantified as the response variable in the analysis, allowing for a comprehensive evaluation of the optimal culture medium.

The culturing process was conducted in Erlenmeyer flasks with a dilution volume of 50 mL, using a 0.1 M phosphate buffer (pH 7), 0.5 % sodium chloride, and 0.2 % dextrose, along with varying concentrations (0.5, 1.0, 1.5, 2.0 g) of each vegetable and animal medium. To facilitate the entry of oxygen, each flask was covered with a cotton plug and gauze. The mixture was sterilized by subjecting it to a temperature of 121 °C for 15 min to ensure its sterility. After the media had been sterilized, the culture was processed following the method described by Bach et al. (2011) [24], Specifically, 100 μL of the bacteria were inoculated into a BHI broth. The Erlenmeyer flasks containing the bacterial culture were then incubated in a thermal bath at 37 °C for three days, with continuous shaking at 180 rpm to promote optimal growth.

#### 2.3.1 Extraction of enzyme extract

At the end of the cultivation time, the enzyme was precipitated using a method adapted from established protocols [25]. Ammonium sulfate was added to each culture medium at a concentration of 60 % w/v and stirred in an ice-cooled thermal bath for 1 h. Afterward, the mixture was refrigerated for an additional hour and subsequently centrifuged at 10,000×*g* for 10 min. The supernatant was decanted, and the resulting precipitate was reconstituted in Tris-HCl buffer at pH 8.

#### 2.3.2 Determination of proteolytic activity

The proteolytic activity in each of the obtained extracts was evaluated using two distinct methods. The first method employed azocasein as the substrate, while the second method utilized casein as the substrate. The protocols for both methodologies were adapted from established procedures found in the literature [26,27], allowing for consistent comparison.

To assess the enzymatic activity using azocasein as the substrate, a reaction mixture was prepared by combining 200 μL of 0.1 M Tris-HCl (pH 8), 200 μL of the extract, and 200 μL of 1 % azocasein. As a reference, a blank solution was created, composed of 200 μL of 1 % azocasein, 1 mL of 10 % trichloroacetic acid (TCA) solution, 200 μL of Tris-HCl, and 200 μL of distilled water. The reaction mixture was incubated at 37 °C for 30 min. To halt the reaction, 1 mL of the TCA solution was added, followed by centrifugation at 5500×*g* for 15 min. After centrifugation, 400 μL of 1.8 N NaOH was introduced to the supernatant, and the absorbance of the resulting solution was measured at a specific wavelength of 420 nm using a UV–Vis 6305 Jenway spectrophotometer.

For the assessment of enzymatic activity using casein as the substrate, 0.1 mL of aqueous extract was placed into two separate test tubes, one for the sample and the other for the blank. The preparation of the blank involved adding 1.8 mL of 10 % trichloroacetic acid (TCA) and 1.1 mL of a 1 % casein solution. To the test tube corresponding to the samples, 1.1 mL of 1 % casein solution was added. Both tubes were then incubated at 37 °C for 20 min. Subsequently, the reaction in the sample tube was stopped by adding 1.8 mL of TCA. Centrifugation was carried out at 3500×*g* for 20 min, followed by the measurement of optical density at 280 nm for both the supernatant of the sample and the blank.

The enzymatic activity was quantified in terms of units (U), where one unit represented the enzyme amount that led to a 0.1 increase in absorbance under the specified assay conditions. The azocaseinolytic and caseinolytic activities were expressed in U/mL of sample for each fermentation run.

### 2.4 Determination of the time of maximum generation of proteolytic activity

To identify the precise time point of maximum proteolytic activity generation, proteolytic activity analyses were conducted at 24-h intervals over a span of 7 days, involving three replicates for each time point. In pursuit of this objective, 300 mL of the optimal growth medium were dispensed into 500 mL Erlenmeyer flasks, maintaining the predetermined conditions [28]. On a daily basis, a volume of 13 mL from each sample was extracted, subsequently undergoing enzymatic precipitation and proteolytic activity assessment utilizing the methods previously outlined.

The determination of the growth kinetics of the microorganism was carried out using the medium that exhibited the highest enzymatic activity. To achieve this, a direct plate count was conducted over a period of 7 days. Exponential dilutions of the selected medium were prepared, ranging from 10^-1 to 10^-11. Inoculation was performed on a sterile Petri dish with 0.1 mL of each dilution, and using an inoculating loop, it was evenly spread across the plate. Incubation was conducted at 37 °C for 48 h in an incubator, followed by the enumeration of microorganisms present on those plates displaying between 30 and 300 colonies.

#### 2.4.1 Purification of enzyme extract

Enzymatic extract purification process was conducted using agarose gel filtration [29]. A column packed with Sephadex G-100 resin, which had been pre-hydrated in 0.1 M phosphate buffer for 24 h, was employed. The column had a diameter of 7.5 mm and a length of 16 cm, with a gel height of 10 cm. Throughout the process, 15 samples were collected for each analysis, with a volume of 1 mL per sample. Subsequently, the caseinolytic and azocaseinolytic activities of the collected samples were assessed to determine the elution volume of the partially purified enzymes.

#### 2.4.2 Determination of the molecular size of the fraction with the highest activity

To determine the molecular weight of the enzymes presents in the collected fractions with the highest proteolytic activity, a polyacrylamide gel electrophoresis analysis was conducted [30]. The hydrolysates were dissolved at a concentration of 20 mg/mL in the loading buffer (50 mM Tris-HCl, 4 % SDS, 12 % glycerol, 2 % mercaptoethanol, and 0.01 % bromophenol blue), and denaturation was carried out at 90 °C for 10 min. Electrophoresis was performed using a Mini Protean II system (Bio-Rad Laboratories, Hercules, CA, USA). A volume of 10 μL was loaded into each lane. Protein bands were stained with brilliant blue Coomassie R250. The approximate molecular weight of the hydrolysates was determined using a molecular weight marker or standard ranging from 20 to 220 kDa in the polyacrylamide gel electrophoresis analysis. A 16.5 % acrylamide gel was used for this purpose. By comparing the migration of the hydrolysates with the known migration of the molecular weight markers, an estimation of their molecular weight can be obtained.

### 2.5 Statistical analysis

The statistical analysis was performed using Statgraphics Centurion XVI software (StatPoint, Inc.). An analysis of variance (ANOVA) was conducted for the analyses, followed by the least significant difference (LSD) procedure with a significance level of p < 0.05.

## 3 Results and discussion

### 3.1 Isolation and identification of spontaneously grown bacteria from the laboratory environment

**Fig. 1:**
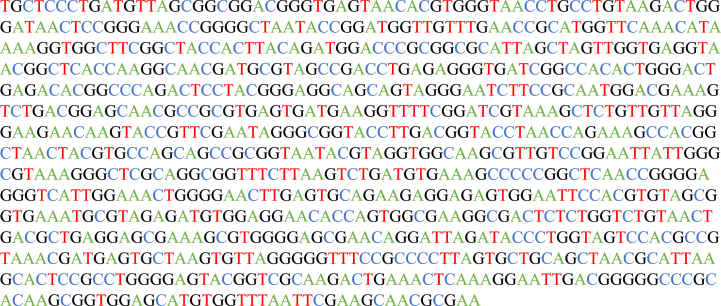
Dna sequence of the isolated microorganism 16S gene.

After 4 days of incubation, a single type of colony was observed, exhibiting uniform shape and size. To ensure consistency, five colonies were randomly selected and subjected to microscopic examination, confirming their identical morphology and distribution pattern. These findings provide compelling evidence of a homogeneous microbial population within the culture. Based on the comprehensive assessment of biochemical and morphological characteristics detailed in Table 1, the isolated strain exhibited discernible attributes. It demonstrated an aerobic metabolism and possessed a rod-shaped morphology, with notable spore-forming capabilities, characteristic of Gram-positive bacteria. The strain also displayed positive motility (Fig. S1a), indicating the presence of flagella. Conversely, the absence of indole production, attributed to the absence of the enzyme tryptophanase responsible for tryptophan degradation, was observed. Moreover, the strain exhibited an inability to utilize citrate as its sole source of energy and carbon (Fig. S1b). It was devoid of urease activity, failing to catalyze the hydrolysis of urea into ammonia, water, and carbon dioxide. Additionally, the strain demonstrated a negative response in the production of hydrogen sulfide (Fig. S1c). Based on these morphological and biochemical attributes, the provisional identification of the microorganism was established as a member of the *Bacillus* genus. Subsequent molecular identification involved the comparison of the 900 bp, nucleotide sequence of the 16S rRNA gene with sequences available in the GenBank database. The sequence of the gene as illustrated in Fig. 1 and Fig. S2. Remarkably, the strain isolated from the gelatin-based medium exhibited 100 % sequence homology with diverse *Bacillus subtilis* strains. The nucleotide sequence of the isolated strain has been deposited and cataloged in the National Center for Biotechnology Information (NCBI) under the unique accession number MH298780. It is important to acknowledge that the coverage provided by a 900 bp DNA sequence may be limited in capturing the complete 16S rRNA gene, and thus, this consideration should be acknowledged as a potential limitation within the context of the identification study.

**Table 1:** Biochemical and morphological characteristics of the isolated strain.

Characteristics	Results
**Colony**	Round and White
**Morphology**	Rods
**Gram staining**	Gram positive
**Endospore staining**	Positive
**Índole**	Negative
**Mobility**	Positive
**Simons Citrate Agar**	Negative
**Urease**	Negative
**H**_**2**_**S production**	Negative

(n = 3).

These analyses were performed according to the specifications in the Bergey's Systematic Manual. The first four characteristics were observed under a microscope, while the remaining five were conducted: Indole (based on tryptophan hydrolysis), Motility (presence of flagella), Simons Citrate Agar (based on citrate metabolism), Urea agar base (based on urease activity), and H2S production (based on hydrogen sulfide production as a result of sulfur compound metabolism).

**Fig. 2:**
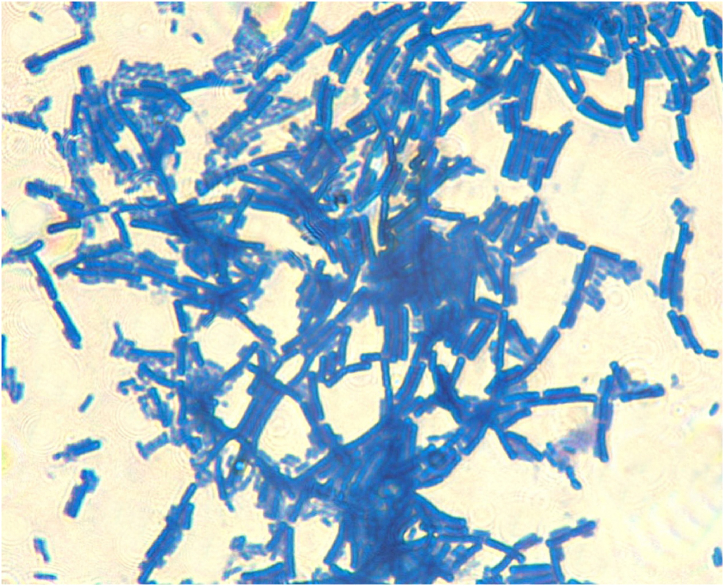
Isolated bacteria using an optical microscope with an attached camera using a Plan *S*-Apo Oil 100X/1.40 Infinity/0.17 lens. The microorganism was dried and stained with methylene blue to observe the endospores (a characteristic feature of Bacillus subtilis).

To provide further evidence supporting the identification of the isolated bacterium as *Bacillus subtilis*, a plate culture was conducted, followed by microscopic examination and imaging (Fig. 2). The captured image reveals the characteristic rod-shaped morphology of *Bacillus subtilis*, with distinct endospores observed at the initial and terminal ends. This observation aligns with the typical features exhibited by *Bacillus subtilis* [31], thus confirming its identification in this study. The microscopic visualization serves as valuable supporting evidence to bolster the argument regarding the presence of *Bacillus subtilis* in the isolated strain. Table 1 shows that the isolated strain exhibits all the necessary biochemical characteristics for gelatin growth. Previous studies have indicated that the gelatin production process is prone to contamination by various thermo-tolerant, aerobic, spore-forming gram-positive and gram-negative bacteria [32,33]. These bacteria can persist even in the final product. The studies mentioned earlier identified four bacterial species: *B. subtilis, B. licheniformis, B. badius, and B. cereus.*

In previous investigations, bacterial strains were isolated from bovine hair and skin waste [33], as well as from gelatin samples obtained from bovine bones and pig skin at different production plants [32]. In both cases, the isolated bacteria were identified as *B. subtilis*. The strain isolated in this study shares similar biochemical and morphological characteristics with the strains studied in the aforementioned references. In the same study, bacterial strains were isolated from bovine hair and skin waste [33], as well as various gelatin samples obtained from bovine bones and pig skin at six production plants [32]. In both cases, the isolated bacteria were identified as *B. subtilis*. The strain isolated in the current study is believed to be *B. subtilis* based on its shared biochemical and morphological characteristics.

Based on the obtained results, the spontaneously grown and subsequently isolated bacteria were identified as *B. subtilis*. Furthermore, it was observed that the use of natamycin did not impact the growth of this bacterial strain. This can be attributed to the absence of sterols in the bacterial membrane. Conversely, fungal membranes, which contain ergosterol, are susceptible to the effects of natamycin [34].

The presence of *Bacillus subtilis* in laboratory settings can be attributed to its ability as a species to produce airborne endospores, which are easily dispersed by air currents. As a result, this bacterium is capable of colonizing diverse environments and initiating growth under suitable conditions. Given its ubiquity, *B. subtilis* demonstrates adaptability to thrive in various ecological niches, including soil, air, water, and the gastrointestinal tracts of animals. Consequently, it can be isolated from a wide range of substrates, reflecting its versatile nature [35]. Therefore, it is likely that this microorganism is present in laboratory environments.

### 3.2 Physicochemical characterization of raw material

The physicochemical analysis of the raw materials revealed that feather meal and soy oil-cake had the highest protein content, with percentages of 91.32 % and 52.63 %, respectively (Table 2). Soy oil-cake, a valuable vegetable-based protein source, has been recognized for its potential in supporting microorganism growth and facilitating efficient enzyme production [36]. Similarly, previous studies have highlighted the suitability of feather meal as a promising raw material for enzyme extraction [37]. Rice polish exhibited the highest percentage of total carbohydrates (74.9 %) and ash content (19.0 %), while fishmeal had lower levels of total carbohydrates (6.7 %) and ash (12.1 %). Additionally, fish and palm kernel oil-cake showed notable concentrations of ethereal extract.

**Table 2:** Proximate analysis for each animal and plant-based culture medium.

	Pa	Ri	So	Bl	Fi	Fe
Protein (%)	16.3 ± 0.04	4.4 ± 0.11	52.6 ± 0.21	82.4 ± 0.19	61.8 ± 0.14	91.3 ± 0.23
Carbohydrates (%)	71.8 ± 0.13	74.9 ± 0.21	37.4 ± 0.18	3.5 ± 0.05	6.7 ± 0.09	2.3 ± 0.2
Ashes (%)	5.5 ± 0.08	19.0 ± 0.13	7.8 ± 0.06	5.4 ± 0.02	12.1 ± 0.04	3.8 ± 0.03
Ether Extract (%)	6.4 ± 0.07	1.8 ± 0.03	2.1 ± 0.01	8.6 ± 0.09	18.3 ± 0.08	2.6 ± 0.06

(n = 3).

Pa = Palm kernel cake, Ri = Rice polish, So = Soybean cake, Bl = Blood meal, Fi = Fish meal, Fe = Feather meal.

Recent research has emphasized the increasing interest in utilizing agro-industrial byproducts with high nutritional value as substrates for microbial protease production [38]. To assess the impact of these byproducts on proteolytic enzyme production, it is essential to comprehend the chemical composition of both animal and plant-based culture media.

### 3.3 Identification of the culture medium for obtaining the extract richest in proteolytic activity

**Fig. 3:**
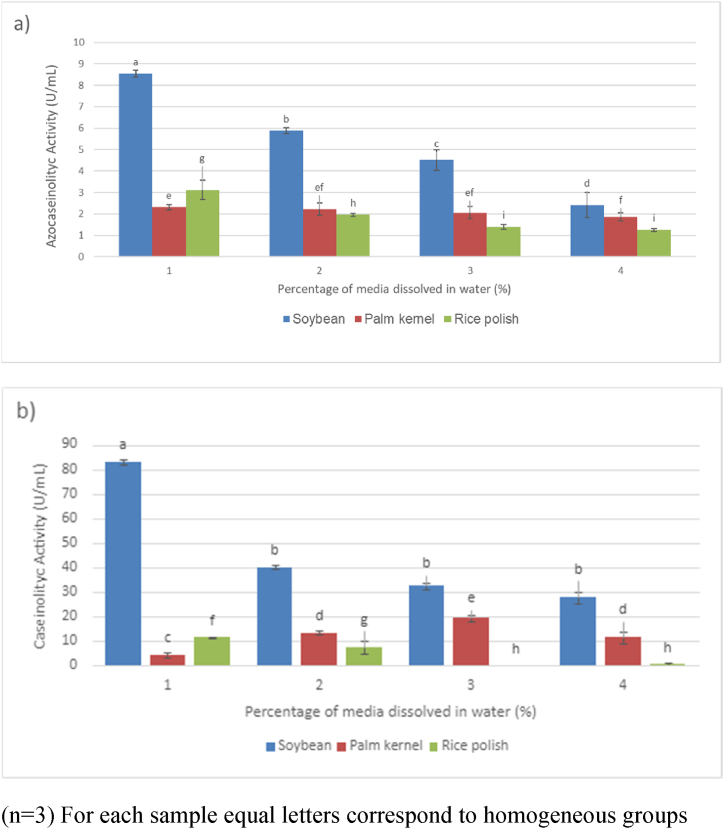
Proteolytic Activity in Plant-Based Media. In Fig. 3a, enzymatic activity is depicted using azocasein as the substrate, while Fig. 3b illustrates the activity using casein as the substrate. In both (a) and (b), the same letter indicates homogeneous groups (p > 0.05). All tests were conducted in triplicate (n = 3). Statistically significant differences among the conducted tests were determined using analysis of variance (ANOVA), followed by a least significant difference (LSD) test at a confidence level of 95 %. Numbers 1, 2, 3 and 4 represent the percentage of media dissolved in water.

Fig. 3a Shows the results of the enzymatic activity after growth in culture media of plant origin, using azocasein as substrate. ANOVA analysis indicated statistically significant differences in enzymatic activity at concentrations of 1 %, 2 %, and 3 % for rice polish and palm kernel oil-cake, as well as at concentrations of 1 % and 4 % for the latter (p < 0.05). However, the proteolytic activities of these two media were relatively low compared to soybean oil-cake.

When analyzing the results obtained with casein (Fig. 3b), the maximum enzymatic activity for palm kernel oil-cake was observed at a concentration of 3 % (19.42 ± 1.31 U/mL). For rice polish, the maximum activity was at a concentration of 1 % (11.54 ± 0.50 U/mL), which decreased with increasing concentration of the culture medium. In contrast, soybean oil-cake at a concentration of 1 % exhibited a statistically significant difference (p < 0.05) in enzymatic activity compared to other concentrations (Fig. 3b). Among all the vegetable media evaluated, soybean oil-cake at a concentration of 1 % displayed the highest azocaseinolytic and caseinolytic activities (Fig. 3a and b). The enzymatic activities were 8.54 ± 0.16 U/mL for azocasein and 83.12 ± 0.12 U/mL for casein. Soybean oil-cake also exhibited the highest protein content among the vegetable substrates, with a value of 52.6 % (Table 2), which correlated with the highest enzymatic activity observed in Fig. 3. Similar findings have been previously reported, demonstrating that substrates with higher protein content lead to increased protease production [39,40]. It was also observed that higher concentrations of the substrate resulted in lower enzymatic activity. This phenomenon has been reported in previous studies, where high concentrations of the culture medium have been found to inhibit protease production due to increased medium viscosity and limited oxygen availability for bacterial growth and enzyme production [41]. It is likely that the isolated strain could not fully metabolize palm kernel oil-cake and rice polish, resulting in lower enzyme production. This could be due to the strain's inability to efficiently utilize these substrates as energy sources, leading to restricted carbon and nitrogen consumption for growth and subsequent secretion of proteolytic enzymes.

Palm kernel oil-cake exhibited an enzymatic activity of 2.31 ± 0.12 U/mL at a concentration of 1 % with azocasein (Fig. 3a). However, this result was not statistically different (p > 0.05) from the other concentrations of this medium, except for the 4 % treatment. Regarding activity with casein as a substrate (Fig. 3b), significant differences were observed at all concentrations, with a maximum of 19.42 ± 1.31 U/mL and a minimum of 4.43 ± 0.81 U/mL.

Analyzing Fig. 3 For rice polish, a similar behavior was observed as with soybean oil-cake, with lower proteolytic activities observed at higher concentrations. This could be attributed to reduced gas/liquid mass transfer in the medium, leading to oxygen limitation and inhibition of enzymatic production by the microorganism [10].

**Fig. 4:**
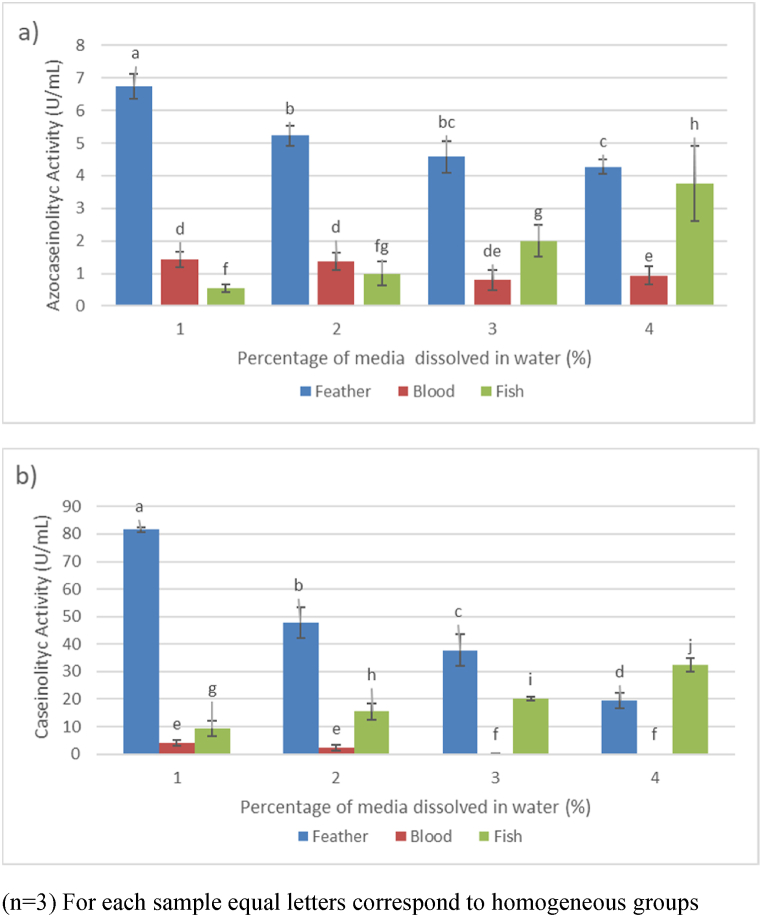
Proteolytic activity for animal-based media. In Fig. 4a, the enzymatic activity is presented using azocasein as the substrate, while Fig. 4b demonstrates the activity using casein as the substrate. Similarly, the use of the same letter indicates homogeneous groups (p > 0.05). All tests were conducted in triplicate (n = 3). Statistically significant differences between the conducted tests were determined using analysis of variance (ANOVA), followed by a least significant difference (LSD) test with a confidence level of 95 %. Numbers 1, 2, 3, and 4 represent the percentages of media dissolved in water.

Regarding animal-based media, Fig. 4a and b demonstrate that feather meal exhibited higher enzymatic activity compared to blood and fish meal. When using feather meal, it was observed that higher substrate percentages resulted in lower enzymatic activity, indicating an inversely proportional relationship between substrate concentration and enzyme production. Previous studies have suggested that increasing feather meal concentration leads to viscosity, inhibiting protease production by the isolated bacteria [42]. Therefore, according to the results of this study, 1 % feather meal is the most suitable animal medium for proteolytic enzyme production. The enzymatic activity observed was 6.75 ± 0.38 U/mL with azocasein and 81.63 ± 0.96 U/mL with casein, using chicken feather meal as a carbon and nitrogen source.

In contrast, when using fish meal as a nutrient medium, a direct relationship was observed between the concentration of the medium and enzyme production (Fig. 4 a and b). Previous research has shown that higher lipid contents in growth media result in greater proteolytic activity [43]. The results strongly indicate a correlation between elevated lipid concentrations and observed enzymatic activity. On the other hand, the presence of high concentrations of carbon sources or carbohydrates in the growth medium can negatively impact enzyme production through carbon-induced repression, where excess carbon inhibits protease synthesis, leading to a decline in proteolytic activity. Consequently, the observed inhibition of proteolytic activity in blood meal samples can be attributed to the substantial carbon content present in the medium. According to previous studies, high concentrations of carbohydrates have a negative effect on enzyme production [44]. However, it has been established that other factors, such as the addition of salts like NaCl, KCl, and MgSO4, can influence protease production [45]. In this study, the addition of these salts promoted protease formation. Therefore, the inclusion of common salt (NaCl) in the media can positively impact enzyme production. The obtained results confirm that the composition of the culture medium significantly affects protease production. Depending on the chemical composition of the substrates, nutritional stress or catabolite repression can occur, inhibiting protease secretion [10].

In relation to the optimal cultivation media identified for enzymatic production in this study, the composition and components of both soybean oil-cake and feather meal play crucial roles in supporting the bacteria's ability to produce proteolytic enzymes. Soybean oil-cake, characterized by its high protein content, serves as an abundant source of amino acids that can be effectively utilized by bacteria for efficient enzyme synthesis. Furthermore, soybean oil-cake encompasses a range of additional substances, including vitamins, sterols, pigments, and organic acids, which have the potential to further enhance the production of enzymes [36]. Similarly, although feather meal exhibits a lower protein content, it still provides essential amino acids and other essential components that effectively support the growth and metabolism of bacteria, enabling them to efficiently produce proteolytic enzymes. These substrates function as valuable carbon and nitrogen sources for bacterial metabolism, facilitating the degradation of complex proteins into smaller peptides and amino acids, which can subsequently be utilized as essential nutrients for bacterial growth and the synthesis of enzymes [46]. Overall, the utilization of both soybean oil-cake and feather meal as cultivation media effectively promotes the production of proteolytic enzymes by providing the necessary nutritional components for bacterial growth and enzymatic activity (Tables S1 and S2).

### 3.4 Time of maximum generation of proteolytic activity in the best media

The determination of the time of maximum generation of proteolytic activity was carried out in the best media, those containing soy oil cake (vegetable medium) and feather meal (animal medium).

**Fig. 5:**
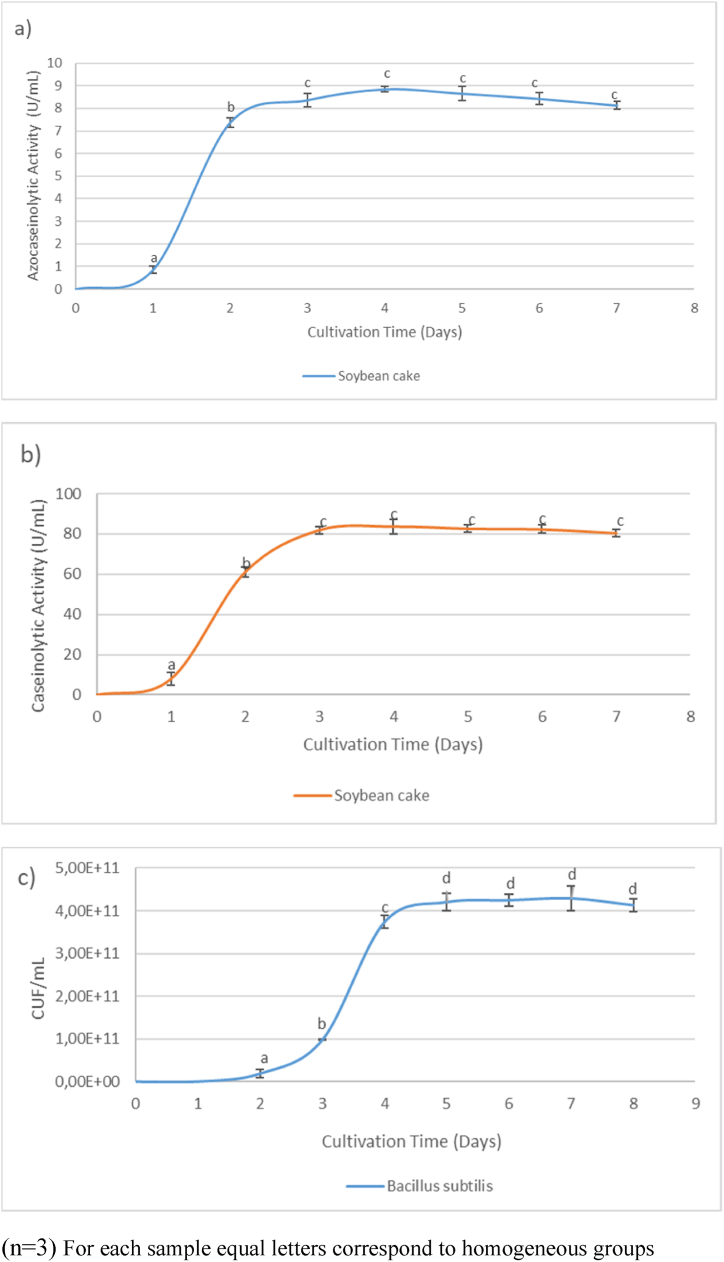
Optimal plant-based culture medium (Soybean cake). Fig. 5a and b illustrate the maximum generation time for enzymatic activity using azocasein and casein as substrates, respectively. Additionally, Fig. 5c depicts bacterial growth in the soybean cake medium. Each assay was performed every 24 h over a period of 7 days, with triplicate measurements (n = 3). Statistically significant differences between the conducted tests were determined using analysis of variance (ANOVA), followed by a least significant difference (LSD) test with a confidence level of 95 %.

Enzymatic activity of soybean oil-cake (Fig. 5a and Table S3a) was evaluated over a period of seven days using azocasein as the substrate. It was observed that proteases were produced from day 1, with the highest activity recorded on day 4 (8.80 U/mL). Similarly, as depicted in Fig. 5b and Table S3b, the maximum activity of 83.71 U/mL was attained on day 4 when casein was used as the substrate. Following day 4, a slight decrease in activity was observed, which could be attributed to autoproteolysis or protease denaturation [28]. Comparatively analyzing the enzymatic production alongside the microbial growth curve reveals a notable pattern: the maximum production of enzymes occurs between the third and fourth day, coinciding with the exponential growth phase of the bacteria. This occurrence can be attributed to the heightened metabolic activity experienced by the bacteria during this phase, resulting in an increased synthesis of proteins, including enzymes [47]. Consequently, a significant portion of the bacterial resources is allocated towards enzyme production, leading to the observed peak in enzymatic activity. Additionally, the exponential growth phase is characterized by ample availability of nutrients, particularly nitrogen sources [48]. Proteases, being crucial for nutrient acquisition and utilization, play a pivotal role in breaking down complex proteins into smaller peptides and amino acids that can be readily assimilated by the bacteria. Thus, the bacteria prioritize protease production during this growth phase to ensure efficient nutrient acquisition and sustained growth. However, following the peak, a subsequent decline in enzyme activity occurs, potentially influenced by factors such as autoproteolysis, where enzymes degrade themselves, or environmental conditions like pH, temperature, or substrate availability, which may contribute to enzyme denaturation or inactivation [49].

Several studies have utilized *B. subtilis* as an enzyme producer [14,28,50]. For instance, maximum protease production was achieved on day 1 using solid waste as a substrate [28], while cassava residues as a protein source led to the highest protease value on day 2 [50]. Similarly, the maximum production was obtained on day 3 in another study [14]. The variations in these results could be attributed to the use of different types of media and conditions, as well as varying inoculum sizes at the beginning, which affected nutrient consumption and oxygen availability. As a result, these factors influenced the duration required to reach maximum protease production. Furthermore, temperature differences may have impacted the production of secondary metabolites.

**Fig. 6:**
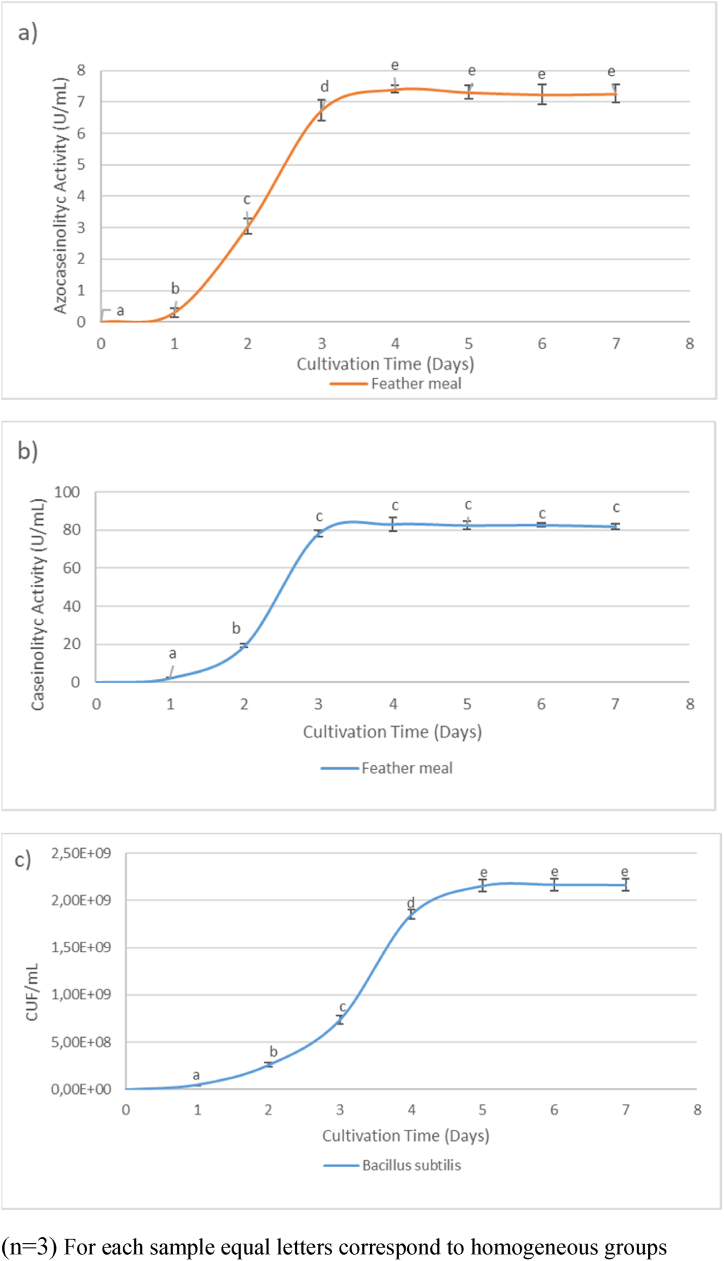
Optimal animal-based culture medium (Feather meal). Fig. 6a–b demonstrate the maximum generation time for enzymatic activity using azocasein and casein as substrates, respectively. Additionally, Fig. 6c portrays bacterial growth in the feather meal medium. Each assay was conducted every 24 h over a period of 7 days, with triplicate measurements (n = 3). Statistically significant differences between the conducted tests were determined using analysis of variance (ANOVA), followed by a least significant difference (LSD) test with a confidence level of 95 %.

Regarding the enzymatic activity of feather meal (Fig. 6), the maximum values were observed on day 4 for both azocasein (7.40 U/mL) and casein (83.1 U/mL) (Fig. 6 a y b, respectively). Similar findings were reported when using fish sauce as a culture medium [51]. However, other studies obtained different results, with maximum production occurring on day five and six [26,51]. These variations could be attributed to differences in the nutritional medium composition and the addition of organic or inorganic supplements. The specific strain used may have also influenced the results, as each microorganism or strain has unique requirements for growth and enzymatic production [35].

The maximum production of proteases in *B. subtilis* can vary from 2 to 9 days, depending on the strain and culture medium employed. In the case of soybean oil-cake and feather meal, their maximum activities were achieved at 4 days. These results indicate that *B. subtilis* is capable of producing proteolytic enzymes using both soybean oil-cake and feather meal as substrates.

In Fig. 5c, the growth curve of Bacillus subtilis in soybean cake is depicted. Exponential growth of the bacterium was observed between days 2 and 4, after which it entered the stationary phase. Similarly, the same pattern is observed when feather meal was used (Fig. 6c), where its exponential phase was observed from day 1 to day 4, after which it initiated its stationary phase. It was observed for this bacterium that both microbial growth kinetics and enzymatic kinetics exhibit a close relationship, reaching their maximum production of proteases when the microorganism is entering its stationary phase.

### 3.5 Purification of enzyme

The fractions collected from the fourth to the sixth day showed notable enzymatic activities (Fig. S3), with the highest activity observed in the fifth fraction of the extracts obtained using soybean oil cake or feather meal. Using the azocasein method (Fig. S3a), the enzymatic activities increased from 8.85 U/mL to 10.91 U/mL for soybean oil-cake, representing a 23.3 % increase, and from 7.40 U/mL to 11.86 U/mL for feather meal, demonstrating a significant 60.9 % increase in activity. Similarly, employing the casein method (Fig. S3b), the activities increased from 83.71 U/mL to 90 U/mL for soybean oil-cake and from 83.10 U/mL to 91.7 U/mL for feather meal, representing percentage increments of 7.5 % and 10.3 %, respectively. These findings confirm the successful generation of a semi-purified enzyme extract, achieved through the elimination of non-enzymatic residues and enzymes with low activity.

### 3.6 Determination of the molecular size of the fractions with the highest enzymatic activity

**Fig. 7:**
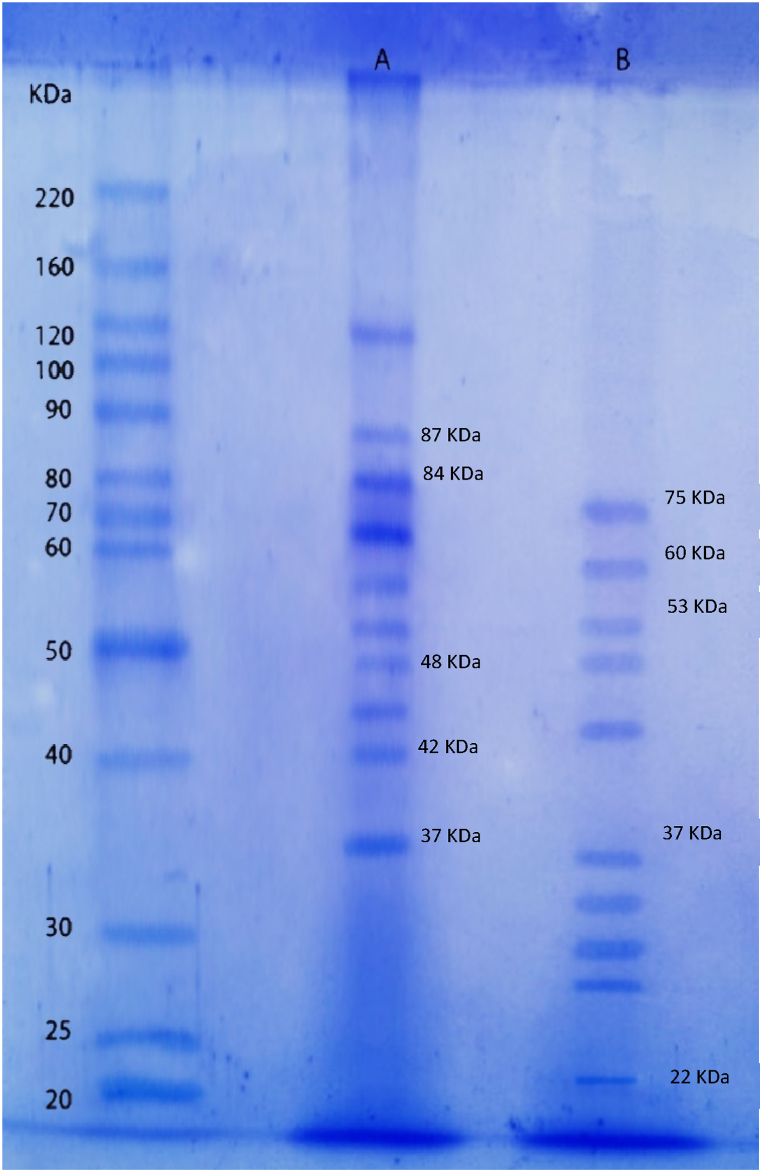
Molecular Weight Sizes on Electrophoresis Gel for the Semipurified Animal (Feather Meal) and Plant (Soybean Cake) Extracts with Higher Enzymatic Activity. In lane a), the bands for the feather meal extract are shown, while in lane b), the bands for the soybean cake extract are presented.

The electrophoresis profile is shown in Fig. 7 and Fig. S4. The lane A (semi-purified extract obtained with the feather meal media) shows several bands of different molecular sizes, the largest enzymatic fraction corresponds to 120 kDa, according to the standard, and the smallest one corresponds to 33 kDa. The lane B shows the profile of the semi-purified extract obtained using the soybean oil cake, with a wide range of molecular sizes that ranged from 75 to 22 kDa. The band of approximately 84 kDa found in lane A may correspond to endopeptidase La (EC 3.4.21.53), according to the NCBI enzyme-Brenda database for *B. subtilis* (Table S4). In this database, 20 records with similar molecular weight have been identified. In addition, there are records of other molecular sizes that also correspond to this enzyme: 87, 60 and 37 kDa. Bands corresponding to these molecular sizes can be observed in lanes A and B.

In previous studies, cow's milk, and a *B. subtilis* strain were used, and three bands corresponding to molecular weights around 42, 48, and 60 kDa were detected. In the semi-purified extract obtained from feather meal, two bands with similar molecular sizes (42 and 48 kDa) can be observed. These molecular weights align with Peptidase Do (EC 3.4.21.107), according to the Brenda enzyme database [52]. Another study reported an enzyme from *B. subtilis* with a molecular weight of 50 kDa, which is similar to the fourth band (4th) observed in the soybean oil-cake lane. The third band obtained exhibits a molecular weight of 53 kDa, corresponding to the *C*-terminal processing peptidase enzyme (EC 3.4.21.102) [53].

According to the NCBI-Brenda database, Signal peptidase (EC 3.4.21.89) produced by *B. subtilis* has a molecular weight of approximately 22 kDa, as observed in lane B. Furthermore, the database registers other enzymes produced by *B. subtilis* within this molecular size, including rhomboid protease (EC 3.4.21.105) and Repressor LexA (3.4.21.82). In both extracts obtained from soybean oil-cake and feather meal, a band of approximately 37 kDa was observed, which could potentially indicate the presence of an alkaline protease according to previous studies [6,54]. However, according to the NCBI enzyme database, the 37 kDa band is attributed to *subtilis*in and endopeptidase La (EC 3.4.21.62 and EC 3.4.21.53).

## 4 Conclusions

The spontaneous growth of aerobic bacteria was observed when utilizing bovine gelatin as a substrate. The microorganisms isolated were identified as Bacillus subtilis. These findings shed light on the potential application of bovine gelatin as a suitable medium for fostering bacterial growth. Moreover, our study delved into the metabolic efficiency of the isolated bacteria across different protein-rich media, highlighting soybean oil-cake and feather meal at a 1 % concentration as the optimal substrates, exhibiting the highest enzymatic activity. Notably, we observed a dose-dependent relationship between culture medium concentration and protease production, emphasizing the need for balanced nutrient provision. Our investigations unveiled that the peak protease production in both soybean oil-cake and feather meal substrates was reached at day 4, employing both azocasein and casein methods for assessment. The semi-purification process of the enzymatic extract obtained from B. subtilis within feather flour and soybean oil-cake unveiled a significant augmentation in azocaseinolytic and caseinolytic activities, underscoring the potential for industrial applications. Furthermore, the electrophoresis gel analysis of fractions exhibiting the highest enzymatic activity in feather flour and soybean oil-cake substrates revealed a multifaceted enzyme profile, suggesting the presence of diverse enzymes with distinct molecular sizes. These insights not only contribute to our understanding of enzymatic processes but also hold implications for advancing research in industrial enzyme production and biotechnological applications in the field.

## Author contribution statement

Alisson Sisa: Performed the experiments, Wrote the paper. Cristina Sotomayor: Conceived and designed the experiments. Lucía Buitrón: Contributed reagents, materials, analysis tools or data. Joaquín Gómez Estaca, Oscar Martínez Alvarez: Analyzed and interpreted the data. Mauricio Mosquera: Conceived and designed the experiments, Analyzed and interpreted the data.

## Funding statement

Dr. Mauricio Esteban Mosquera was supported by Escuela Politécnica Nacional {PIJ 16–12}.

Oscar Martinez was suported by the Spanish National Research Council (CSIC, Spain) under project iCOOPA22013.

## Data availability statement

Data will be made available on request.

## Declaration of competing interest

The authors declare that they have no known competing financial interests or personal relationships that could have appeared to influence the work reported in this paper.
