# Supplementary material for: Evaluation of by-products from agricultural, livestock and fishing industries as nutrient source for the production of proteolytic enzymes
Source: Heliyon. 2023 Oct 10;9(10):e20735. doi: 10.1016/j.heliyon.2023.e20735 (PMC10585220; doi:10.1016/j.heliyon.2023.e20735)
Supplement: Multimedia component 1 [file mmc1.docx]

**Supplementary Material**

**Figure S1**. Biochemical and morphological tests of bacteria isolated from gelatin. A) Positive motility test (flagellum) of the isolated bacterium: A tear is observed in the culture medium, indicating that the microorganism had mobility through the flagellum. Negative indole test of the isolated bacterium: There was no fuchsia ring on the surface of the culture broth. B) Simons Citrate Agar test negative for isolated bacteria in the culture medium, monoammonium phosphate is the only source of nitrogen and sodium citrate is the only source of carbon, both components are necessary for bacterial growth. If the bacterium can use citrate as the only source of carbon and energy, the medium will turn blue, and this is indicative of the production of citrate permease. C) Negative H2S test of the isolated bacterium: The black precipitate in the background formed by iron salts did not appear.

**Figure S2.** Chromatogram depicting the 16S gene of the isolated microorganism obtained from the environment, cultured in a medium of bovine gelatin at a temperature of 37°C.

**Figure S3.** Partial purification curve of enzymes obtained from soybean oil cake and feather meal, depicting their purification progress concerning the number of collected fractions. The purification process utilized a column filled with Sephadex G-100 resin, with each fraction containing 1 mL of the sample. Subplot (a) illustrates the azocaseinolytic activity, while subplot (b) displays the caseinolytic activity.

**Figure S4.** Molecular Weight Sizes on Electrophoresis Gel for the Semipurified Animal (Feather Meal) and Plant (Soybean Cake) Extracts with Higher Enzymatic Activity. In lane a), the bands for the feather meal extract are shown, while in lane b), the bands for the soybean cake extract are presented.

**Table S1.** Evaluation of Proteolytic Activity across Various Substrates: Soybean Oil Cake, Palm Kernel Oil Cake, Rice Polish, Feather Meal, Bovine Blood Meal, and Fish Meal, Using Azocaseinolytic Activity as an Assessment Method.

**Table S2.** Evaluation of Proteolytic Activity across Various Substrates: Soybean Oil Cake, Palm Kernel Oil Cake, Rice Polish, Feather Meal, Bovine Blood Meal, and Fish Meal, Using Caseinolytic Activity as an Assessment Method.

**Table S3.** Time of maximum generation of enzymatic activity for the best culture media using azocasein as substrate.

**Table S4.** Proteases produced by Bacillus subtilis registered in the BRENDA database of the ncbi. Yellow Color: Molecular Sizes for Feather Meal; Blue Color: Molecular Sizes for Soybean Cake; Green color: Molecular sizes for both substrates


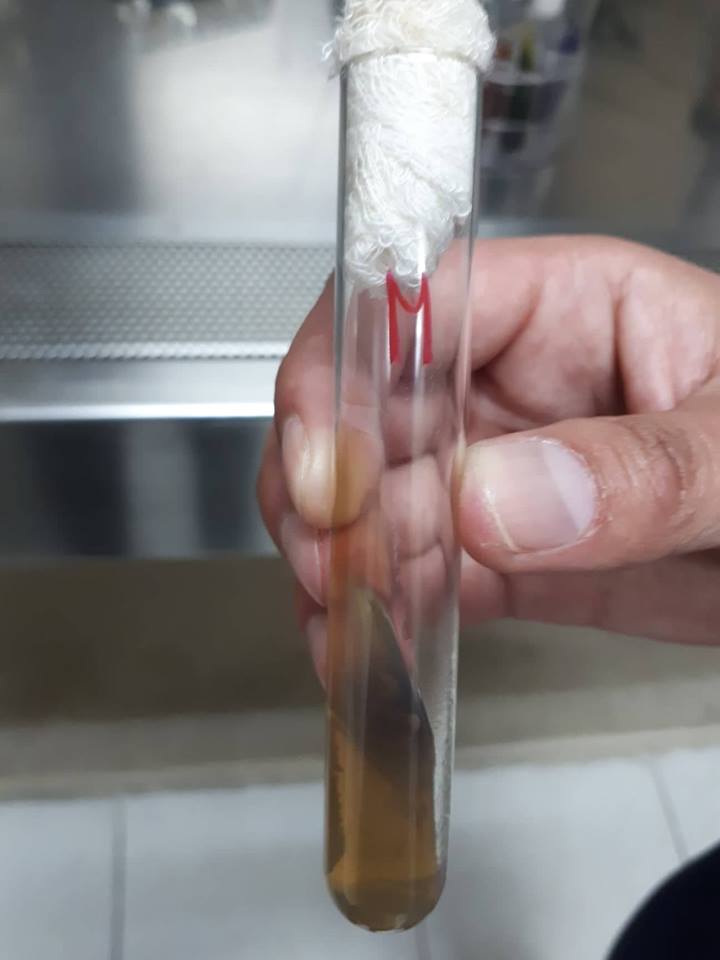

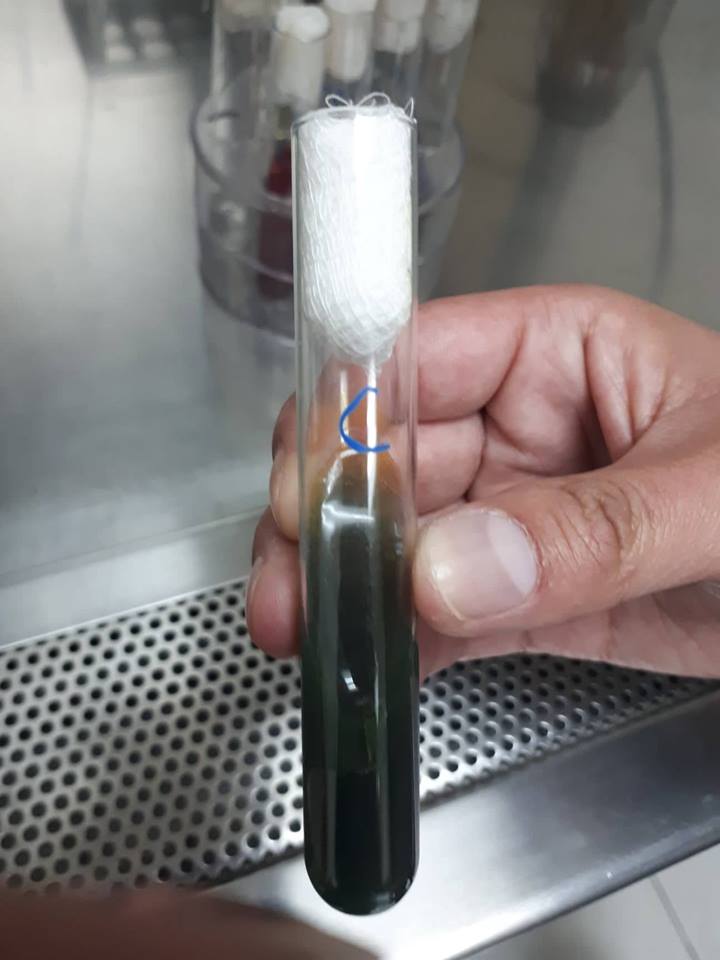

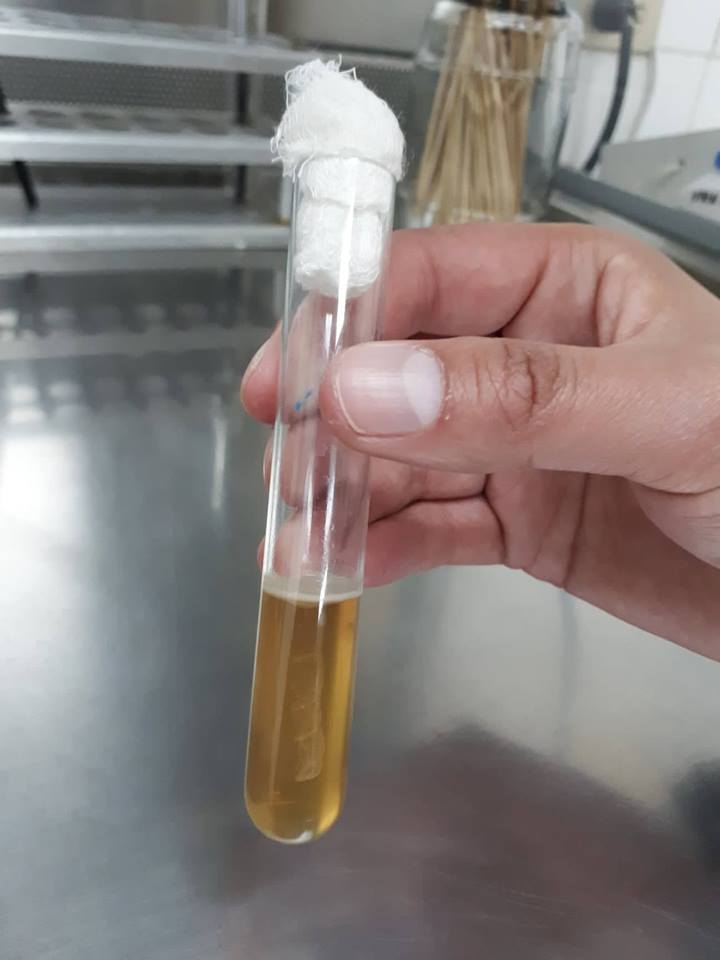


C

B

A

**Figure S1**. Biochemical and morphological tests of bacteria isolated from gelatin. A) Positive motility test (flagellum) of the isolated bacterium: A tear is observed in the culture medium, indicating that the microorganism had mobility through the flagellum. Negative indole test of the isolated bacterium: There was no fuchsia ring on the surface of the culture broth. B) Simons Citrate Agar test negative for isolated bacteria in the culture medium, monoammonium phosphate is the only source of nitrogen and sodium citrate is the only source of carbon, both components are necessary for bacterial growth. If the bacterium can use citrate as the only source of carbon and energy, the medium will turn blue, and this is indicative of the production of citrate permease. C) Negative H2S test of the isolated bacterium: The black precipitate in the background formed by iron salts did not appear.

|

**Figure S2.** Chromatogram depicting the 16S gene of the isolated microorganism obtained from the environment, cultured in a medium of bovine gelatin at a temperature of 37°C.


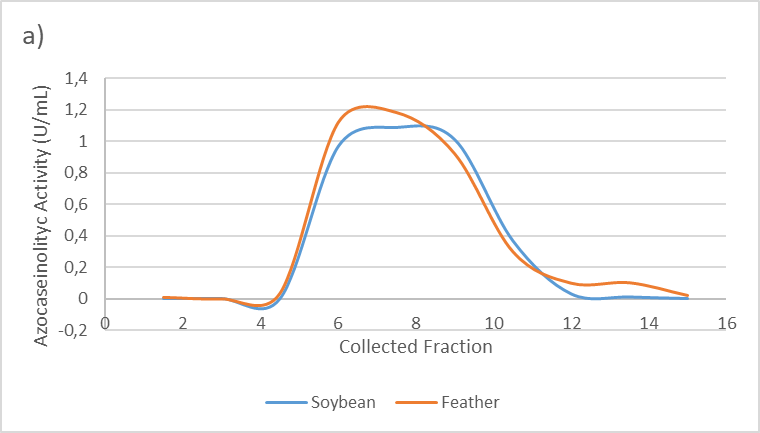


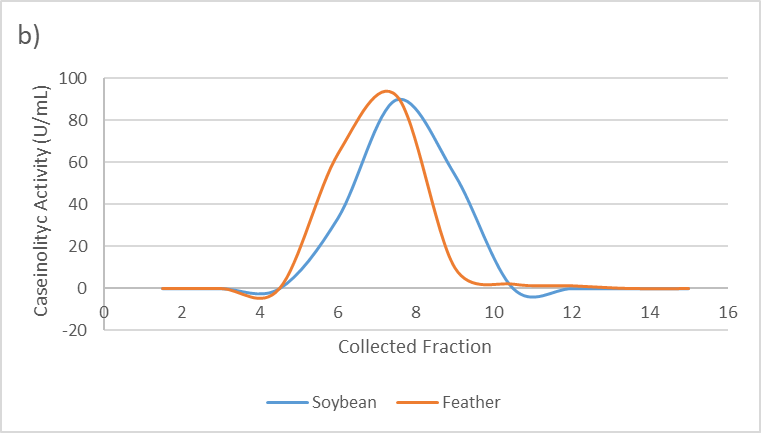


**Figure S3.** Partial purification curve of enzymes obtained from soybean oil cake and feather meal, depicting their purification progress concerning the number of collected fractions. The purification process utilized a column filled with Sephadex G-100 resin, with each fraction containing 1 mL of the sample. Subplot (a) illustrates the azocaseinolytic activity, while subplot (b) displays the caseinolytic activity.

**
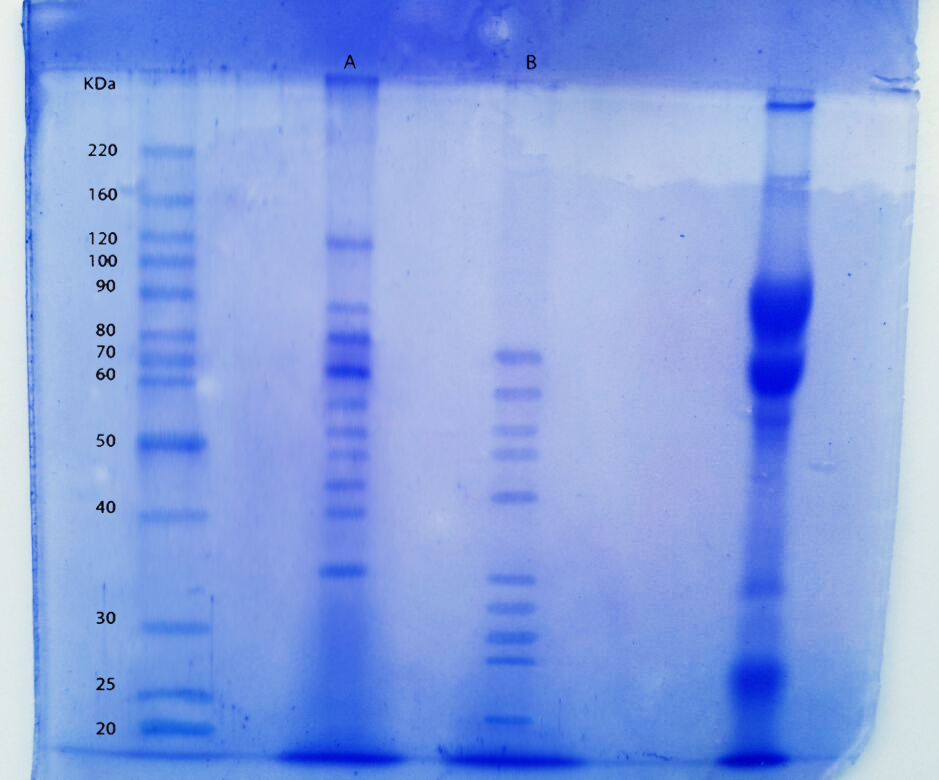
**

87 KDa

48 KDa

42 KDa

53 KDa

60 KDa

75 KDa

22 KDa

37 KDa

84 KDa

37 KDa

**Figure S4.** Molecular Weight Sizes on Electrophoresis Gel for the Semipurified Animal (Feather Meal) and Plant (Soybean Cake) Extracts with Higher Enzymatic Activity. In lane a), the bands for the feather meal extract are shown, while in lane b), the bands for the soybean cake extract are presented.

**Table S1.** Evaluation of Proteolytic Activity across Various Substrates: Soybean Oil Cake, Palm Kernel Oil Cake, Rice Polish, Feather Meal, Bovine Blood Meal, and Fish Meal, Using Azocaseinolytic Activity as an Assessment Method.

| **Sample** | **1%** | **2%** | **3%** | **4%** |
| --- | --- | --- | --- | --- |
| Soybean oil-cake | ${8.54\pm0.16}^{a}$ | ${5.89 \pm0.13}^{b}$ | ${4.51 \pm0.47}^{c}$ | ${2.41\pm0.59}^{d}$ |
| Palm kernel oil-cake | ${2.31\pm0.12}^{e}$ | ${2.2\pm0.28}^{ef}$ | ${2.00\pm0.28}^{ef}$ | ${1.86\pm0.19}^{f}$ |
| Rice polish | ${3.12\pm0.46}^{g}$ | ${1.96\pm0.06}^{h}$ | ${1.39 \pm0.11}^{i}$ | ${1.25 \pm0.06}^{i}$ |
| Feather meal | ${6.75\pm0.38}^{a}$ | ${5.23 \pm0.32}^{b}$ | ${4.50\pm0.48}^{bc}$ | ${4.27 \pm0.22}^{c}$ |
| Bovine blood meal | ${1.42\pm0.23}^{d}$ | ${1.37\pm0.27}^{d}$ | ${0.8 \pm0.31}^{de}$ | ${0.94 \pm0.28}^{e}$ |
| Fish meal | ${0.54\pm0.13}^{f}$ | ${1.0\pm0.37}^{fg}$ | ${2.00\pm0.49}^{g}$ | ${3.75\pm1.15}^{h}$ |

x ± 𝞼. The mean and standard deviation are shown (n = 3). For each sample, the same letters in the rows correspond to homogeneous groups.

**Table S2.** Evaluation of Proteolytic Activity across Various Substrates: Soybean Oil Cake, Palm Kernel Oil Cake, Rice Polish, Feather Meal, Bovine Blood Meal, and Fish Meal, Using Caseinolytic Activity as an Assessment Method.

| **Sample** | **1%** | **2%** | **3%** | **4%** |
| --- | --- | --- | --- | --- |
| Rice polish | ${11.54\pm0.50}^{f}$ | ${7.53 \pm2,73}^{g}$ | ${0,00 \pm0,00}^{h}$ | ${0.70 \pm0.62}^{h}$ |
| Soybean oil-cake | ${83.12\pm0.12}^{a}$ | ${40.27\pm1.37}^{b}$ | ${32.67\pm4.33}^{b}$ | ${27.98\pm7.54}^{b}$ |
| Palm kernel oil-cake | ${4.43 \pm0.81}^{c}$ | ${13.57\pm0.67}^{d}$ | ${19.42 \pm1.31}^{e}$ | ${11.57\pm2.30}^{d}$ |
| Feather meal | ${81.63 \pm0.96}^{a}$ | ${47.91 \pm5.56}^{b}$ | ${37.82 \pm5.89}^{c}$ | ${19.40 \pm2.85}^{d}$ |
| Bovine blood meal | ${4.1 \pm1.13}^{e}$ | ${2.35 \pm1.03}^{e}$ | ${0.27\pm0.10}^{f}$ | ${0.00 \pm0.00}^{f}$ |
| Fish meal | ${9.33 \pm2.93}^{g}$ | ${15.52 \pm2.97}^{h}$ | ${20.22 \pm0.70}^{i}$ | ${32.28\pm2.38}^{j}$ |

x ± 𝞼. The mean and standard deviation are shown (n = 3). For each sample, the same letters in the rows correspond to homogeneous groups.

**Table S3** Time of Maximum Enzymatic Activity Generation for Optimal Culture Media Using Azocasein as Substrate: A) Soybean oil-cake B) Feather meal. For this measurement, the sample was not subjected to purification.

**A)**

| **TIME**  **(days)** | **Sample 1**  **(U/mL)** | **Sample 2**  **(U/mL)** | **Sample 3**  **(U/mL)** | **Mean**  **(U/mL)** | ± 𝞼 |
| --- | --- | --- | --- | --- | --- |
| 0 | 0.00 | 0.00 | 0.00 | 0.00 | 0.00 |
| 1 | 0.98 | 0.678 | 0.88 | 0.84 | 0.15 |
| 2 | 7.15 | 7.58 | 7.40 | 7.37 | 0.21 |
| 3 | 8.16 | 8.25 | 8.72 | 8.38 | 0.30 |
| 4 | 8.79 | 8.99 | 8.79 | 8.86 | 0.11 |
| 5 | 8.33 | 8.72 | 8.94 | 8.66 | 0.30 |
| 6 | 8.15 | 8.54 | 8.61 | 8.43 | 0.24 |
| 7 | 7.94 | 8.17 | 8.31 | 8.14 | 0.18 |

**B)**

| **TIME**  **(days)** | **Sample 1**  **(nm)** | **Sample 2**  **(nm)** | **Sample 3**  **(nm)** | **Mean**  **(nm)** | ± 𝞼 |
| --- | --- | --- | --- | --- | --- |
| 0 | 0.00 | 0.00 | 0.00 | 0.00 | 0.00 |
| 1 | 0.15 | 0.45 | 0.30 | 0.30 | 0.15 |
| 2 | 3.26 | 3.09 | 2.79 | 3.04 | 0.23 |
| 3 | 7.03 | 6.39 | 6.78 | 6.73 | 0.32 |
| 4 | 7.30 | 7.52 | 7.38 | 7.40 | 0.11 |
| 5 | 7.29 | 7.52 | 7.10 | 7.30 | 0.21 |
| 6 | 7.58 | 7.14 | 6.98 | 7.23 | 0.31 |
| 7 | 7.39 | 7.45 | 6.93 | 7.26 | 0.28 |

**Table S4.** Proteases produced by Bacillus subtilis registered in the BRENDA database of the ncbi

Yellow Color: Molecular Sizes for Feather Meal

Blue Color: Molecular Sizes for Soybean Cake

Green color: Molecular sizes for both substrates

| **EC Number** | **Recommended Name** | **Organism** | **UNIPROT** | **No of amino acids** | **MW [Da]** | **Source** |
| --- | --- | --- | --- | --- | --- | --- |
| 3.4.21.19 | glutamyl endopeptidase | Bacillus subtilis subsp. subtilis | A0A1Y0U002 | 313 amino acids | 33856 | TrEMBL |
| 3.4.21.53 | Endopeptidase La | Bacillus subtilis subsp. inaquosorum KCTC 13429 | L8Q1S9 | 774 amino acids | 86712 | TrEMBL |
| 3.4.21.53 | Endopeptidase La | Bacillus subtilis | A0A1L3PVJ4 | 774 amino acids | 86695 | TrEMBL |
| 3.4.21.53 | Endopeptidase La | Bacillus subtilis subsp. spizizenii (strain TU-B-10) | G4NYP0 | 774 amino acids | 86675 | TrEMBL |
| 3.4.21.53 | Endopeptidase La | Bacillus subtilis | A0A0D1JKX6 | 774 amino acids | 86671 | TrEMBL |
| 3.4.21.53 | Endopeptidase La | Bacillus subtilis | A0A0A0TS06 | 774 amino acids | 86665 | TrEMBL |
| 3.4.21.53 | Endopeptidase La | Bacillus subtilis subsp. subtilis | A0A1Y0UIM2 | 774 amino acids | 86648 | TrEMBL |
| 3.4.21.53 | Endopeptidase La | Bacillus subtilis | A0A136GC12 | 774 amino acids | 86630 | TrEMBL |
| 3.4.21.53 | Endopeptidase La | Bacillus subtilis subsp. spizizenii (strain ATCC 23059 / NRRL B-14472 / W23) | E0TVA0 | 774 amino acids | 86625 | TrEMBL |
| 3.4.21.53 | Endopeptidase La | Bacillus subtilis XF-1 | M4KU18 | 774 amino acids | 86621 | TrEMBL |
| 3.4.21.53 | Endopeptidase La | Bacillus subtilis | A0A162TEI5 | 774 amino acids | 86616 | TrEMBL |
| 3.4.21.53 | Endopeptidase La | Bacillus subtilis (strain 168) | P37945 | 774 amino acids | 86607 | Swiss-Prot |
| 3.4.21.53 | Endopeptidase La | Bacillus subtilis BEST7613 | L8APC5 | 774 amino acids | 86607 | TrEMBL |
| 3.4.21.53 | Endopeptidase La | Bacillus subtilis subsp. subtilis str. SC-8 | G4ESU4 | 774 amino acids | 86607 | TrEMBL |
| 3.4.21.53 | Endopeptidase La | Bacillus subtilis subsp. subtilis | A0A199WJI3 | 774 amino acids | 86607 | TrEMBL |
| 3.4.21.53 | Endopeptidase La | Bacillus subtilis | A0A063XIY5 | 774 amino acids | 86607 | TrEMBL |
| 3.4.21.53 | Endopeptidase La | Bacillus subtilis subsp. natto (strain BEST195) | D4FZM9 | 774 amino acids | 86598 | TrEMBL |
| 3.4.21.53 | Endopeptidase La | Bacillus subtilis | A0A1A0G627 | 774 amino acids | 86596 | TrEMBL |
| 3.4.21.53 | Endopeptidase La | Bacillus subtilis | A0A1G4LM17 | 774 amino acids | 86572 | TrEMBL |
| 3.4.21.53 | Endopeptidase La | Bacillus subtilis | A0A0K6MXQ3 | 776 amino acids | 86505 | TrEMBL |
| 3.4.21.53 | Endopeptidase La | Bacillus subtilis subsp. niger | A0A080UM82 | 774 amino acids | 86352 | TrEMBL |
| 3.4.21.53 | Endopeptidase La | Bacillus subtilis | A0A0K6MX31 | 557 amino acids | 61603 | TrEMBL |
| 3.4.21.53 | Endopeptidase La | Bacillus subtilis | A0A0D1KWW4 | 552 amino acids | 60430 | TrEMBL |
| 3.4.21.53 | Endopeptidase La | Bacillus subtilis (strain 168) | P42425 | 552 amino acids | 60429 | Swiss-Prot |
| 3.4.21.53 | Endopeptidase La | Bacillus subtilis subsp. natto (strain BEST195) | D4FZN0 | 552 amino acids | 60429 | TrEMBL |
| 3.4.21.53 | Endopeptidase La | Bacillus subtilis subsp. subtilis | A0A1B2B5V2 | 552 amino acids | 60429 | TrEMBL |
| 3.4.21.53 | Endopeptidase La | Bacillus subtilis | A0A164WWT8 | 552 amino acids | 60429 | TrEMBL |
| 3.4.21.53 | Endopeptidase La | Bacillus subtilis BEST7613 | L8ANW4 | 552 amino acids | 60429 | TrEMBL |
| 3.4.21.53 | Endopeptidase La | Bacillus subtilis subsp. inaquosorum KCTC 13429 | L8Q0N4 | 552 amino acids | 60422 | TrEMBL |
| 3.4.21.53 | Endopeptidase La | Bacillus subtilis XF-1 | M4KUF0 | 552 amino acids | 60402 | TrEMBL |
| 3.4.21.53 | Endopeptidase La | Bacillus subtilis | A0A1A0G621 | 552 amino acids | 60393 | TrEMBL |
| 3.4.21.53 | Endopeptidase La | Bacillus subtilis subsp. subtilis str. SC-8 | G4ESU3 | 552 amino acids | 60389 | TrEMBL |
| 3.4.21.53 | Endopeptidase La | Bacillus subtilis | A0A1L3PVK0 | 552 amino acids | 60375 | TrEMBL |
| 3.4.21.53 | Endopeptidase La | Bacillus subtilis subsp. spizizenii (strain TU-B-10) | G4NYP1 | 552 amino acids | 60370 | TrEMBL |
| 3.4.21.53 | Endopeptidase La | Bacillus subtilis | A0A1G4LM64 | 552 amino acids | 60365 | TrEMBL |
| 3.4.21.53 | Endopeptidase La | Bacillus subtilis subsp. spizizenii (strain ATCC 23059 / NRRL B-14472 / W23) | E0TVA1 | 552 amino acids | 60361 | TrEMBL |
| 3.4.21.53 | Endopeptidase La | Bacillus subtilis | A0A136GC71 | 552 amino acids | 60327 | TrEMBL |
| 3.4.21.53 | Endopeptidase La | Bacillus subtilis subsp. niger | A0A080UI74 | 552 amino acids | 60297 | TrEMBL |
| 3.4.21.53 | Endopeptidase La | Bacillus subtilis | A0A0A0TZP6 | 552 amino acids | 60290 | TrEMBL |
| 3.4.21.53 | Endopeptidase La | Bacillus subtilis | A0A164ZXX8 | 341 amino acids | 37652 | TrEMBL |
| 3.4.21.53 | Endopeptidase La | Bacillus subtilis BEST7613 | L8AKU9 | 341 amino acids | 37651 | TrEMBL |
| 3.4.21.53 | Endopeptidase La | Bacillus subtilis | A0A063XGQ7 | 341 amino acids | 37651 | TrEMBL |
| 3.4.21.53 | Endopeptidase La | Bacillus subtilis subsp. subtilis | A0A199WH06 | 341 amino acids | 37651 | TrEMBL |
| 3.4.21.53 | Endopeptidase La | Bacillus subtilis | A0A1A0GAP9 | 341 amino acids | 37629 | TrEMBL |
| 3.4.21.53 | Endopeptidase La | Bacillus subtilis | A0A136G6Q0 | 341 amino acids | 37613 | TrEMBL |
| 3.4.21.53 | Endopeptidase La | Bacillus subtilis subsp. spizizenii (strain TU-B-10) | G4NS28 | 341 amino acids | 37598 | TrEMBL |
| 3.4.21.53 | Endopeptidase La | Bacillus subtilis subsp. inaquosorum KCTC 13429 | L8PYZ8 | 341 amino acids | 37572 | TrEMBL |
| 3.4.21.53 | Endopeptidase La | Bacillus subtilis subsp. spizizenii (strain ATCC 23059 / NRRL B-14472 / W23) | E0U4K1 | 341 amino acids | 37541 | TrEMBL |
| 3.4.21.53 | Endopeptidase La | Bacillus subtilis | A0A0A0TQ06 | 340 amino acids | 37480 | TrEMBL |
| 3.4.21.53 | Endopeptidase La | Bacillus subtilis | A0A0K6MMQ5 | 340 amino acids | 37426 | TrEMBL |
| 3.4.21.53 | Endopeptidase La | Bacillus subtilis subsp. niger | A0A080UVR2 | 340 amino acids | 37358 | TrEMBL |
| 3.4.21.53 | Endopeptidase La | Bacillus subtilis | A0A1L3PS83 | 340 amino acids | 37324 | TrEMBL |
| 3.4.21.53 | Endopeptidase La | Bacillus subtilis | A0A0D1L2E1 | 332 amino acids | 36437 | TrEMBL |
| 3.4.21.53 | Endopeptidase La | Bacillus subtilis subsp. subtilis str. SC-8 | G4EWP2 | 332 amino acids | 36437 | TrEMBL |
| 3.4.21.53 | Endopeptidase La | Bacillus subtilis subsp. natto (strain BEST195) | D4FW99 | 332 amino acids | 36437 | TrEMBL |
| 3.4.21.53 | Endopeptidase La | Bacillus subtilis XF-1 | M4KY31 | 331 amino acids | 36337 | TrEMBL |
| 3.4.21.62 | Subtilisin | Bacillus subtilis | A0A1L2BPI2 | 381 amino acids | 39579 | TrEMBL |
| 3.4.21.62 | Subtilisin | Bacillus subtilis XF-1 | M4KWV6 | 381 amino acids | 39576 | TrEMBL |
| 3.4.21.62 | Subtilisin | Bacillus subtilis subsp. spizizenii (strain TU-B-10) | G4NRS4 | 381 amino acids | 39573 | TrEMBL |
| 3.4.21.62 | Subtilisin | Bacillus subtilis | Q84F18 | 381 amino acids | 39555 | TrEMBL |
| 3.4.21.62 | Subtilisin | Bacillus subtilis subsp. natto | P35835 | 381 amino acids | 39507 | Swiss-Prot |
| 3.4.21.62 | Subtilisin | Bacillus subtilis | A0A1L2BPI4 | 381 amino acids | 39495 | TrEMBL |
| 3.4.21.62 | Subtilisin | Bacillus subtilis (strain 168) | P04189 | 381 amino acids | 39479 | Swiss-Prot |
| 3.4.21.62 | Subtilisin | Bacillus subtilis subsp. subtilis str. SC-8 | G4EY69 | 381 amino acids | 39479 | TrEMBL |
| 3.4.21.62 | Subtilisin | Bacillus subtilis subsp. amylosacchariticus | P00783 | 381 amino acids | 39467 | Swiss-Prot |
| 3.4.21.62 | Subtilisin | Bacillus subtilis | A0A1L2BPI3 | 381 amino acids | 39462 | TrEMBL |
| 3.4.21.62 | Subtilisin | Bacillus subtilis | A0A1L2BPI7 | 381 amino acids | 39459 | TrEMBL |
| 3.4.21.62 | Subtilisin | Bacillus subtilis subsp. subtilis | A0A1Y0UEB0 | 381 amino acids | 39451 | TrEMBL |
| 3.4.21.62 | Subtilisin | Bacillus subtilis | B6VFQ8 | 381 amino acids | 39437 | TrEMBL |
| 3.4.21.62 | Subtilisin | Bacillus subtilis | X2JEV8 | 381 amino acids | 39433 | TrEMBL |
| 3.4.21.62 | Subtilisin | Bacillus subtilis | A0A024B5N4 | 381 amino acids | 39419 | TrEMBL |
| 3.4.21.62 | Subtilisin | Bacillus subtilis | X5DBC3 | 381 amino acids | 39407 | TrEMBL |
| 3.4.21.62 | Subtilisin | Bacillus subtilis | X5D4R3 | 381 amino acids | 39405 | TrEMBL |
| 3.4.21.62 | Subtilisin | Bacillus subtilis subsp. niger | A0A080UK04 | 382 amino acids | 39206 | TrEMBL |
| 3.4.21.62 | Subtilisin | Bacillus subtilis | A0A024B5D8 | 382 amino acids | 39170 | TrEMBL |
| 3.4.21.62 | Subtilisin | Bacillus subtilis | A0A1L2BPI0 | 360 amino acids | 37038 | TrEMBL |
| 3.4.21.62 | Subtilisin | Bacillus subtilis | B0FXJ2 | 354 amino acids | 35922 | TrEMBL |
| 3.4.21.62 | Subtilisin | Bacillus subtilis | A0A0K6MFU0 | 297 amino acids | 31929 | TrEMBL |
| 3.4.21.88 | Repressor LexA | Bacillus subtilis | A0A0K6N2A1 | 218 amino acids | 24391 | TrEMBL |
| 3.4.21.88 | Repressor LexA | Bacillus subtilis subsp. natto (strain BEST195) | D4FX88 | 208 amino acids | 23265 | TrEMBL |
| 3.4.21.88 | Repressor LexA | Bacillus subtilis subsp. subtilis str. SC-8 | G4EVU0 | 208 amino acids | 23265 | TrEMBL |
| 3.4.21.88 | Repressor LexA | Bacillus subtilis | A0A086WWY6 | 206 amino acids | 23047 | TrEMBL |
| 3.4.21.88 | Repressor LexA | Bacillus subtilis subsp. niger | A0A080UK08 | 206 amino acids | 22979 | TrEMBL |
| 3.4.21.88 | Repressor LexA | Bacillus subtilis | A0A0K6MKH7 | 206 amino acids | 22890 | TrEMBL |
| 3.4.21.88 | Repressor LexA | Bacillus subtilis subsp. spizizenii (strain ATCC 23059 / NRRL B-14472 / W23) | E0TVV5 | 205 amino acids | 22861 | TrEMBL |
| 3.4.21.88 | Repressor LexA | Bacillus subtilis (strain 168) | P31080 | 205 amino acids | 22849 | Swiss-Prot |
| 3.4.21.88 | Repressor LexA | Bacillus subtilis | A0A063XAX5 | 205 amino acids | 22849 | TrEMBL |
| 3.4.21.88 | Repressor LexA | Bacillus subtilis XF-1 | M4KVK4 | 205 amino acids | 22849 | TrEMBL |
| 3.4.21.89 | Signal peptidase I | Bacillus subtilis BEST7613 | L8AHB8 | 218 amino acids | 24733 | TrEMBL |
| 3.4.21.89 | Signal peptidase I | Bacillus subtilis BEST7613 | L8ACL3 | 196 amino acids | 22230 | TrEMBL |
| 3.4.21.89 | Signal peptidase I | Bacillus subtilis subsp. niger | A0A080ULE3 | 193 amino acids | 21938 | TrEMBL |
| 3.4.21.89 | Signal peptidase I | Bacillus subtilis | A0A1A0GCG3 | 193 amino acids | 21882 | TrEMBL |
| 3.4.21.89 | Signal peptidase I | Bacillus subtilis subsp. spizizenii (strain ATCC 23059 / NRRL B-14472 / W23) | E0U3V8 | 193 amino acids | 21880 | TrEMBL |
| 3.4.21.89 | Signal peptidase I | Bacillus subtilis subsp. spizizenii (strain TU-B-10) | G4NWM2 | 193 amino acids | 21868 | TrEMBL |
| 3.4.21.89 | Signal peptidase I | Bacillus subtilis subsp. inaquosorum KCTC 13429 | L8PXB9 | 193 amino acids | 21868 | TrEMBL |
| 3.4.21.89 | Signal peptidase I | Bacillus subtilis | A0A0K6LIG2 | 193 amino acids | 21868 | TrEMBL |
| 3.4.21.89 | Signal peptidase I | Bacillus subtilis | A0A086X2N6 | 193 amino acids | 21860 | TrEMBL |
| 3.4.21.89 | Signal peptidase I | Bacillus subtilis (strain 168) | P71013 | 193 amino acids | 21854 | Swiss-Prot |
| 3.4.21.102 | C-terminal processing peptidase | Bacillus subtilis | A0A0K6MI44 | 494 amino acids | 54024 | TrEMBL |
| 3.4.21.102 | C-terminal processing peptidase | Bacillus subtilis subsp. niger | A0A080UGQ9 | 483 amino acids | 53065 | TrEMBL |
| 3.4.21.102 | C-terminal processing peptidase | Bacillus subtilis subsp. subtilis | A0A1Y0UF49 | 480 amino acids | 52826 | TrEMBL |
| 3.4.21.102 | C-terminal processing peptidase | Bacillus subtilis (strain 168) | O35002 | 480 amino acids | 52798 | Swiss-Prot |
| 3.4.21.102 | C-terminal processing peptidase | Bacillus subtilis subsp. spizizenii (strain TU-B-10) | G4P1A6 | 480 amino acids | 52762 | TrEMBL |
| 3.4.21.102 | C-terminal processing peptidase | Bacillus subtilis | A0A1G4LEJ8 | 480 amino acids | 52728 | TrEMBL |
| 3.4.21.102 | C-terminal processing peptidase | Bacillus subtilis subsp. subtilis | A0A1Y0U8U3 | 473 amino acids | 52158 | TrEMBL |
| 3.4.21.102 | C-terminal processing peptidase | Bacillus subtilis (strain 168) | O34666 | 466 amino acids | 51149 | Swiss-Prot |
| 3.4.21.102 | C-terminal processing peptidase | Bacillus subtilis subsp. subtilis str. SC-8 | G4EUX7 | 466 amino acids | 51149 | TrEMBL |
| 3.4.21.102 | C-terminal processing peptidase | Bacillus subtilis subsp. subtilis | A0A1Y0UC43 | 466 amino acids | 51120 | TrEMBL |
| 3.4.21.102 | C-terminal processing peptidase | Bacillus subtilis subsp. spizizenii (strain TU-B-10) | G4NSC3 | 466 amino acids | 50956 | TrEMBL |
| 3.4.21.105 | rhomboid protease | Bacillus subtilis subsp. subtilis | A0A1Y0U5V9 | 507 amino acids | 56604 | TrEMBL |
| 3.4.21.105 | rhomboid protease | Bacillus subtilis subsp. niger | A0A080UNB8 | 511 amino acids | 56592 | TrEMBL |
| 3.4.21.105 | rhomboid protease | Bacillus subtilis (strain 168) | P54493 | 507 amino acids | 56462 | Swiss-Prot |
| 3.4.21.105 | rhomboid protease | Bacillus subtilis subsp. spizizenii (strain ATCC 23059 / NRRL B-14472 / W23) | E0U436 | 506 amino acids | 56371 | TrEMBL |
| 3.4.21.105 | rhomboid protease | Bacillus subtilis subsp. spizizenii (strain ATCC 23059 / NRRL B-14472 / W23) | E0U436 | 506 amino acids | 56371 | TrEMBL |
| 3.4.21.105 | rhomboid protease | Bacillus subtilis subsp. subtilis | A0A1Y0U020 | 199 amino acids | 22471 | TrEMBL |
| 3.4.21.105 | rhomboid protease | Bacillus subtilis | A0A0K6MGU8 | 190 amino acids | 21288 | TrEMBL |
| 3.4.21.107 | peptidase Do | Bacillus subtilis subsp. subtilis | A0A1Y0UB44 | 458 amino acids | 48746 | TrEMBL |
| 3.4.21.107 | peptidase Do | Bacillus subtilis (strain 168) | Q9R9I1 | 458 amino acids | 48717 | Swiss-Prot |
| 3.4.21.107 | peptidase Do | Bacillus subtilis subsp. subtilis | A0A1Y0TXP5 | 453 amino acids | 48137 | TrEMBL |
| 3.4.21.107 | peptidase Do | Bacillus subtilis (strain 168) | O34358 | 449 amino acids | 47715 | Swiss-Prot |
| 3.4.21.107 | peptidase Do | Bacillus subtilis | A0A0K6MEC4 | 413 amino acids | 43869 | TrEMBL |
| 3.4.21.107 | peptidase Do | Bacillus subtilis subsp. subtilis | A0A1Y0UQD8 | 400 amino acids | 42756 | TrEMBL |
| 3.4.21.107 | peptidase Do | Bacillus subtilis | A0A0K6MES2 | 391 amino acids | 41888 | TrEMBL |
| 3.4.21.116 | SpoIVB peptidase | Bacillus subtilis | A0A0K6MTE1 | 432 amino acids | 46958 | TrEMBL |
| 3.4.21.116 | SpoIVB peptidase | Bacillus subtilis subsp. niger | A0A080UJ85 | 426 amino acids | 46168 | TrEMBL |
| 3.4.21.116 | SpoIVB peptidase | Bacillus subtilis subsp. subtilis | A0A1Y0U6B2 | 426 amino acids | 46085 | TrEMBL |
| 3.4.21.116 | SpoIVB peptidase | Bacillus subtilis (strain 168) | P17896 | 426 amino acids | 46075 | Swiss-Prot |
| 3.4.21.116 | SpoIVB peptidase | Bacillus subtilis (strain 168) | P26937 | 288 amino acids | 33640 | Swiss-Prot |
| 3.4.21.116 | SpoIVB peptidase | Bacillus subtilis (strain 168) | P26937 | 288 amino acids | 33640 | Swiss-Prot |
| 6.3.4.21 | nicotinate phosphoribosyltransferase | Bacillus subtilis | A0A0K6MFL2 | 487 amino acids | 56293 | TrEMBL |
| 6.3.4.21 | nicotinate phosphoribosyltransferase | Bacillus subtilis | A0A0K6LDL2 | 490 amino acids | 56221 | TrEMBL |
| 6.3.4.21 | nicotinate phosphoribosyltransferase | Bacillus subtilis subsp. spizizenii (strain TU-B-10) | G4P064 | 490 amino acids | 56221 | TrEMBL |
| 6.3.4.21 | nicotinate phosphoribosyltransferase | Bacillus subtilis | A0A0C3LWJ9 | 490 amino acids | 56214 | TrEMBL |
| 6.3.4.21 | nicotinate phosphoribosyltransferase | Bacillus subtilis subsp. natto (strain BEST195) | D4G0R9 | 490 amino acids | 56208 | TrEMBL |
| 6.3.4.21 | nicotinate phosphoribosyltransferase | Bacillus subtilis subsp. spizizenii (strain ATCC 23059 / NRRL B-14472 / W23) | E0TZW8 | 490 amino acids | 56193 | TrEMBL |
| 6.3.4.21 | nicotinate phosphoribosyltransferase | Bacillus subtilis | A0A1A0FQM4 | 490 amino acids | 56187 | TrEMBL |
| 6.3.4.21 | nicotinate phosphoribosyltransferase | Bacillus subtilis (strain 168) | O32090 | 490 amino acids | 56180 | Swiss-Prot |
| 6.3.4.21 | nicotinate phosphoribosyltransferase | Bacillus subtilis subsp. | G4ERS0 | 490 amino acids | 56180 | TrEMBL |
